# A duodenal tumor near the major duodenal papilla treated using a new thin therapeutic endoscope following pancreatic and biliary stenting

**DOI:** 10.1055/a-2717-1736

**Published:** 2025-10-29

**Authors:** Hitoshi Mori, Koh Kitagawa, Satoshi Iwai, Yukihisa Fujinaga, Akira Mitoro, Hitoshi Yoshiji

**Affiliations:** 1 12967 Department of Gastroenterology, Nara Medical University, Nara, Japan; 2 12967 Division of Endoscopy, Nara Medical University, Nara, Japan

Duodenal endoscopic submucosal dissection (ESD) is technically challenging due to the complex anatomy of the duodenum 1 . In particular, when the lesion is close to the major duodenal papilla, the risk of pancreatitis and cholangitis increases. A recently introduced thin therapeutic endoscope (EG-840TP; Fujifilm, Tokyo, Japan) is reportedly useful for performing ESD in anatomically narrow spaces ( Fig. 1 ) 2 3 .

**Fig. 1:**
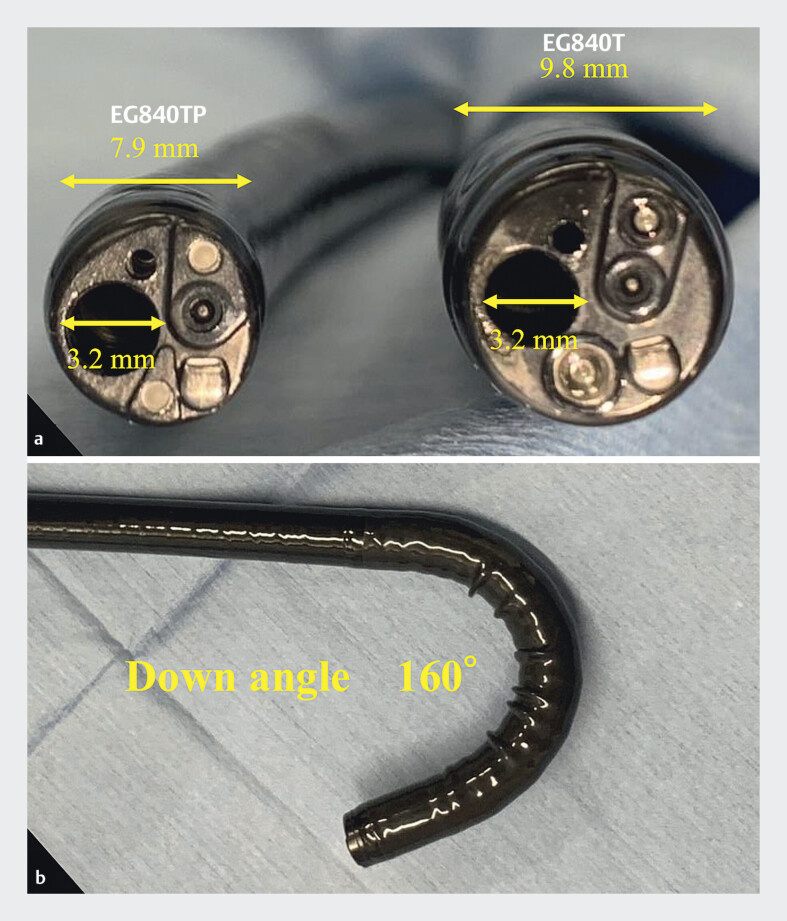
**a** Specifications of the thin therapeutic endoscope. It has an accessory channel with a diameter of 3.2 mm and an outer diameter of only 7.9 mm. **b** The thin therapeutic endoscope allows an extended downward angulation of up to 160°.

Herein, we report a case of duodenal ESD performed with a novel thin endoscope following pancreatic and biliary stenting. A 77-year-old woman was diagnosed with a 20-mm in diameter duodenal tumor located near the major duodenal papilla and was referred to our hospital for endoscopic treatment ( Fig. 2 **a** ). First, pancreatic stenting and biliary stenting were performed to minimize the risk of thermal damage to the orifice of the major duodenal papilla ( Fig. 2 **b** ). Subsequently, ESD was performed using the thin therapeutic endoscope ( Fig. 2 **c** ). To prevent cholangitis and pancreatitis after ESD caused by air insufflation, the water exchange method was used. The lesion was completely resected using ESD, and the mucosal defect was closed using the reopenable-clip over-the-line method ( Fig. 2 **d, e** , Video 1 ) 4 . On the day following the procedure, the patient developed mild acute pancreatitis, which quickly resolved with conservative treatment. The pancreatic duct stent was endoscopically removed on postoperative day 7, and the patient was discharged 8 days after the duodenal ESD. The final pathological diagnosis was high-grade adenoma with negative margins. Two months later, the biliary stent had passed spontaneously. This new thin therapeutic endoscope has a 3.2-mm accessory channel, a waterjet function, and an extended down angle of up to 160°. Therefore, it is useful for treating lesions near the major duodenal papilla, even when pancreatic stenting and biliary stenting are in place.

**Fig. 2:**
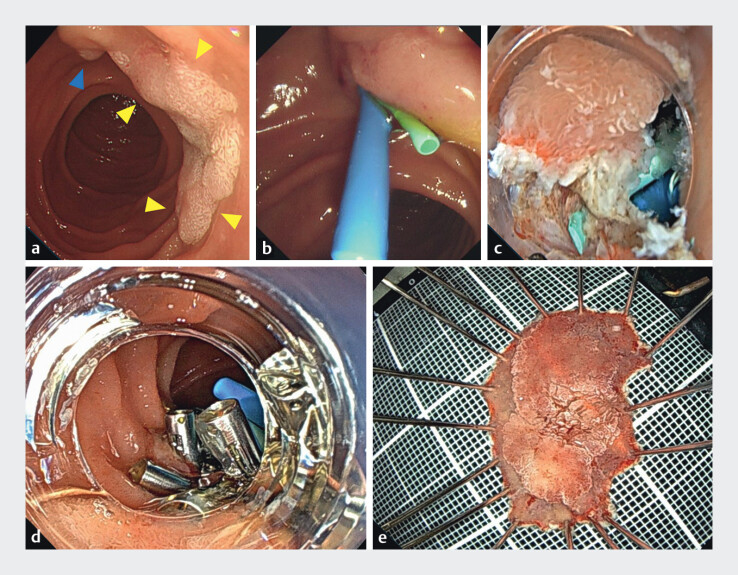
**a** Endoscopic view of the lesion. A Paris IIa 20 mm in diameter non-ampullary duodenal adenoma (yellow arrowheads) located near the major papilla (blue arrowhead). Endoscopic findings suggested the possibility of an adenoma. **b** Endoscopic retrograde cholangiopancreatography was performed. Pancreatic and biliary plastic stents were placed before endoscopic submucosal dissection to prevent thermal injury to the orifice of the major duodenal papilla. **c** Endoscopic submucosal dissection was performed using a thin therapeutic endoscope fitted with a small-caliber transparent tip hood (DH-083ST; Fujifilm). **d** Mucosal defect closure using the reopenable-clip over the line method. **e** Resected specimen.

A duodenal tumor near the major duodenal papilla treated using a new thin therapeutic endoscope following pancreatic and biliary stenting.Video 1

Endoscopy_UCTN_Code_TTT_1AO_2AG_3AD

## Notes

Endoscopy E-Videos https://eref.thieme.de/e-videos
*E-Videos* is an open access online section of the journal *Endoscopy* , reporting on interesting cases and new techniques in gastroenterological endoscopy. All papers include a high-quality video and are published with a Creative Commons CC-BY license. Endoscopy E-Videos qualify for HINARI discounts and waivers and eligibility is automatically checked during the submission process. We grant 100% waivers to articles whose corresponding authors are based in Group A countries and 50% waivers to those who are based in Group B countries as classified by Research4Life (see: https://www.research4life.org/access/eligibility/ ). This section has its own submission website at https://mc.manuscriptcentral.com/e-videos .
